# Association Between Multiple Inflammation‐Derived Indices and the Risk of Post‐Stroke Epilepsy

**DOI:** 10.1002/cns.71143

**Published:** 2026-09-08

**Authors:** Yu Wang, YeQin Gao, Long Wang

**Affiliations:** ^1^ Graduate School of Wan Nan Medical College Wuhu Anhui China; ^2^ Department of Neurology The Second People's Hospital of Hefei Hefei Anhui China; ^3^ Anhui Medical University Hefei Anhui China; ^4^ The Fifth Clinical School of Medicine, Anhui Medical University Hefei Anhui China

**Keywords:** post‐stroke epilepsy, propensity score matching, risk prediction, systemic immune‐inflammation index

## Abstract

**Background:**

Post‐stroke epilepsy (PSE) is a common and serious complication of stroke, yet simple and efficient risk assessment tools remain lacking in clinical practice. This study aimed to investigate the association between multiple core inflammation‐derived indices and PSE, and to explore their potential reference value in risk stratification.

**Methods:**

In this retrospective case–control study, we extracted data from hospitalized stroke patients at Hefei Second People's Hospital between January 2018 and December 2025 from the electronic medical record system. A 1:1 propensity score matching method was used to balance confounding factors including sex, age, and underlying diseases. Ultimately, 317 PSE patients (case group) and 317 stroke patients without PSE (control group) were included. Routine hematological parameters collected within 24 h of admission were used to calculate six inflammatory indices: the systemic immune‐inflammation index (SIRI), neutrophil‐to‐lymphocyte ratio (NLR), derived neutrophil‐to‐lymphocyte ratio (DNLR), monocyte‐to‐lymphocyte ratio (MLR), neutrophil‐to‐platelet ratio (NPR), and neutrophil–monocyte‐to‐lymphocyte ratio (NMLR). Multivariable logistic regression models were employed to analyze the association between inflammatory indices and PSE risk, and nonlinear relationships and threshold effects were also explored. The discriminative ability and incremental value of each inflammatory index were assessed using receiver operating characteristic (ROC) curves, decision curve analysis (DCA), net reclassification improvement (NRI), and integrated discrimination improvement (IDI). Subgroup analyses were conducted to verify the stability of the results.

**Results:**

This study showed that for each one‐standard‐deviation increase in SIRI, the risk of PSE increased by 172% (OR = 2.72, FDR‐adjusted *p* < 0.001). Quartile‐stratified analysis revealed a clear dose–response relationship (trend *p* < 0.001). Similarly, the strength of association for SIRI was superior to that of other inflammation‐derived indices. Compared with the other inflammatory indices, SIRI demonstrated relatively better discriminative ability in ROC analysis (AUC = 0.693, 95% CI: 0.654–0.732), NRI, and DCA. Restricted cubic spline regression and *E*‐value analysis further supported the robustness of this association, and subgroup analyses indicated consistency across different subgroups.

**Conclusion:**

Systemic immune‐inflammation index showed a relatively strong association with PSE risk and moderate discriminative ability in the stroke population, and may serve as an auxiliary reference indicator for early risk stratification of post‐stroke epilepsy. However, its clinical utility requires further validation in prospective cohort studies.

## Graphical abstract

This propensity score‐matched case–control study (*n* = 634) evaluated six inflammation‐derived indices for post‐stroke epilepsy risk stratification. SIRI demonstrated the strongest association (OR = 2.72, 95% CI: 2.04–3.63), moderate discriminative ability (AUC = 0.693), and robustness to unmeasured confounding (*E*‐value = 4.88).

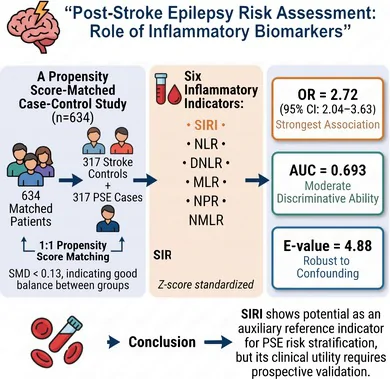

## 1 Introduction

Stroke is the second leading cause of death from noncommunicable diseases worldwide and the third leading cause of disability‐adjusted life years (DALYs). Its prevalence and mortality show a continuous upward trend, and the scale of the disabled population is expanding [1]. With the improvement of clinical treatment for stroke, the patient survival rate has been significantly improved, but the clinical management of post‐stroke complications has become an urgent problem [2]. The incidence of early‐onset seizure after stroke is 2%–20%, and the incidence of post‐stroke epilepsy (PSE) is approximately 6%–15% [3]. This complication not only aggravates neurological impairment in patients but also imposes a heavy medical and economic burden on patients' families and society. Therefore, early identification of high‐risk populations for PSE and the exploration of novel, simple, and efficient risk assessment indicators are of significant clinical importance for guiding interventions, reducing PSE incidence, and improving long‐term patient outcomes.

The occurrence of PSE is closely related to long‐term structural brain changes and abnormal blood–brain barrier stability. The pathological mechanisms involve multiple processes such as glial scar formation, glial cell activation, and hemosiderin deposition [4, 5, 6, 7, 8]. These pathological changes can lead to secondary cerebral edema, which further increases the risk of epileptic seizures due to insufficient cerebral perfusion. At the same time, the central role of the inflammatory response in the occurrence and development of PSE has been confirmed by numerous studies. Hu et al. found that the baseline systemic immune‐inflammation index (SIRI) level in PSE patients was significantly higher than that in non‐PSE patients. After covariate adjustment, SIRI was still significantly positively correlated with PSE occurrence (OR = 1.99, *p* < 0.001), with an obvious dose–response relationship [9]. Another study confirmed that the neutrophil/lymphocyte ratio (NLR) was significantly associated with the risk of epilepsy after intracerebral hemorrhage [10, 11]. In addition, the regulatory roles of inflammation‐related factors such as IL‐1*β*, IL‐6, and TNF‐*α* in PSE have gradually become research hotspots [12, 13, 14]. Inflammation‐derived indexes based on routine blood parameters have the advantages of easy availability, low cost, and rapid detection, and have become an important research direction for PSE risk prediction. However, systematic studies on the association between multiple inflammation‐derived indices and PSE, as well as their risk assessment value, remain lacking, and the threshold effects, discriminative ability, and clinical utility of different indices are still unclear.

In the field of PSE risk assessment, the classic SeLECT model incorporates risk factors including stroke severity and large artery atherosclerosis etiology to construct a scoring system [15]. Subsequent researchers integrated acute symptomatic seizure types to develop the SeLECT 2.0 score [16]. Tomotaka Tanaka et al. incorporated cortical superficial siderosis (cSS) into the SeLECT and CAVE scores, and the established SeLECT‐S and CAVE‐S models improved PSE risk assessment performance [17]. Other investigators adjusted the weighting of the CAVE score and obtained the CAVE^2^ score with higher discriminative accuracy [18]. However, most existing PSE risk assessment models are primarily based on clinical and imaging features, lacking systematic integration and application of inflammation‐derived indices. Moreover, some models suffer from limitations such as small sample sizes and insufficient external validation, making it difficult to meet the clinical requirements for simple and rapid PSE risk stratification.

Therefore, this study systematically investigated the association between six inflammation‐derived indices based on routine blood parameters (including SIRI, NLR, and DNLR) and the risk of PSE. Multivariable logistic regression was applied to determine the strength of association and dose–response relationships between the indices and PSE. Restricted cubic spline analysis was used to explore nonlinear relationships and threshold effects. ROC curves and DCA were combined to comprehensively evaluate the discriminative ability and net clinical benefit of each index for PSE. In addition, statistical evaluations were performed using NRI, IDI, and subgroup analyses. This study aimed to preliminarily explore the association between inflammation‐derived indices and PSE risk and their potential reference value in risk stratification, thereby optimizing clinical management strategies for stroke patients.

## 2 Methods

### 2.1 Study Design and Study Population

**FIGURE 1:**
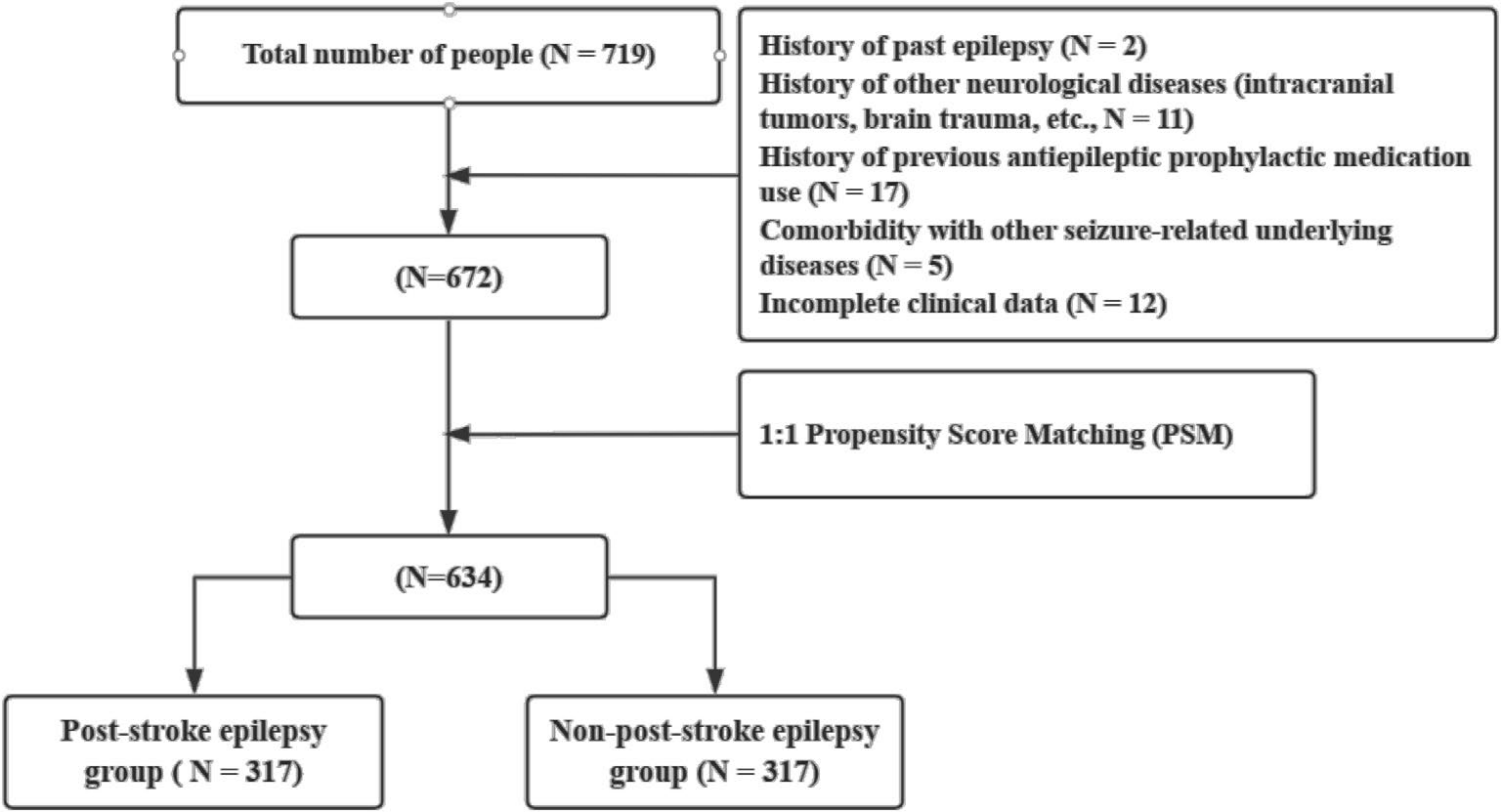
Participant Flowchart. PSM, propensity score matching; PSE, post‐stroke epilepsy. The flowchart illustrates the process of patient selection and 1:1 propensity score matching in this study.

A retrospective case–control study design was employed. Electronic medical record data were extracted from stroke patients hospitalized at Hefei Second People's Hospital between January 2018 and December 2025. Based on the occurrence of PSE, a case group and a control group were established, and 1:1 propensity score matching (PSM) was performed. The propensity score model included 11 variables. Logistic regression was used to estimate propensity scores, and nearest‐neighbor matching was applied for 1:1 matching, with the caliper set at 0.2 times the standard deviation of the propensity score, without replacement. After matching, a total of 634 patients were successfully matched (317 controls and 317 cases). Covariate balance before and after matching was assessed using standardized mean differences (SMDs) (Table S2, Figures S1, S2). The case group included 317 patients diagnosed with cerebral infarction or intracerebral hemorrhage by cranial computed tomography (CT) or magnetic resonance imaging (MRI) who met the diagnostic criteria for unprovoked seizures occurring more than 7 days after stroke [19, 20, 21, 22]. The control group consisted of 317 stroke patients admitted to the same hospital during the same period who were diagnosed with cerebral infarction or intracerebral hemorrhage by the same imaging modalities and who did not experience unprovoked seizures within 7 days after stroke. Using the stroke diagnosis time of case patients as the anchor, potential confounding factors including sex, age, and underlying diseases such as hypertension, diabetes mellitus, and coronary heart disease were matched to ensure comparability of key clinical characteristics between the two groups (Figure 1).

### 2.2 Definition and Calculation of Inflammatory Indicators

Six core inflammation‐derived indexes were included in this study, all calculated based on routine hematological parameters detected within 24 h after admission. All tests were performed by the hospital laboratory using standardized procedures. The specific calculation formulas were as follows: Systemic immune‐inflammation index (SIRI) = Absolute neutrophil count (ANC) × Absolute monocyte count (AMC)/Absolute lymphocyte count (ALC); Neutrophil‐to‐lymphocyte ratio (NLR) = Absolute neutrophil count (ANC)/Absolute lymphocyte count (ALC); Derived neutrophil‐to‐lymphocyte ratio (DNLR) = Absolute neutrophil count (ANC)/(White blood cell count (WBC) − Absolute neutrophil count (ANC)); Monocyte‐to‐lymphocyte ratio (MLR) = Absolute monocyte count (AMC)/Absolute lymphocyte count (ALC); Neutrophil‐to‐platelet ratio (NPR) = Absolute neutrophil count (ANC)/Platelet count (PLT); Neutrophil–monocyte to lymphocyte ratio (NMLR) = (Absolute neutrophil count (ANC) + Absolute monocyte count (AMC))/Absolute lymphocyte count (ALC). All inflammatory indicators were standardized by *Z*‐score to eliminate dimensional differences for subsequent nonlinear relationship analysis and threshold effect analysis.

### 2.3 Covariate Collection

All variables were collected and adjudicated by uniformly trained neurologists using standardized methods to ensure data consistency. Demographic and underlying disease indicators were obtained through face‐to‐face structured interviews combined with electronic medical record reviews, including age, sex, smoking history, drinking history, and confirmed histories of hypertension, diabetes mellitus, cerebral infarction, and coronary heart disease. BMI (kg/m^2^) was calculated from weight measured using a standard scale and height measured using a stadiometer; systolic blood pressure (SBP, mmHg) and diastolic blood pressure (DBP, mmHg) were measured using an electronic sphygmomanometer on the right upper arm after the patient had rested for 15 min, with the average of three consecutive measurements recorded. Laboratory parameters were all derived from fasting venous blood samples collected within 24 h of admission and tested by the hospital laboratory according to standardized procedures. Electrolytes (potassium, sodium) were measured using ion‐selective electrode methods; glycated hemoglobin was measured using high‐performance liquid chromatography; renal function indicators (urea, creatinine, uric acid) were measured using enzymatic or colorimetric methods; *β*2‐microglobulin was measured using immunoturbidimetry; hematological parameters (white blood cell count, red blood cell count, hemoglobin, platelet count, neutrophil count, lymphocyte count, monocyte count) were measured using an automated hematology analyzer; liver function indicators (alanine aminotransferase, aspartate aminotransferase, albumin) were measured using rate methods or bromocresol green methods; lipid metabolism indicators (triglycerides, total cholesterol) were measured using enzymatic methods; and homocysteine was measured using the enzymatic cycling method. In addition, patients were divided into two subgroups according to the time from stroke onset to admission blood sampling: ≤ 6 h and 6–24 h. Furthermore, we extracted clinical variables including cortical involvement (confirmed by CT or MRI reports) and early seizures (seizures occurring within 7 days after stroke) from electronic medical records and imaging reports.

### 2.4 Statistical Methods

All statistical analyses in this study were performed using R software (version 4.5.2), and a two‐sided *p* < 0.05 was considered statistically significant. In the data preprocessing phase, multiple imputation was used to handle missing values; covariates with a missing rate < 10% were imputed. For baseline characteristic comparisons, categorical variables were expressed as numbers (percentages) [*n* (%)] and compared between groups using the chi‐square test; continuous variables following a normal distribution were described as mean ± standard deviation (Mean ± SD) and compared using independent samples *t*‐tests; continuous variables not following a normal distribution were described as median (interquartile range) [M (Q_1_, Q_3_)] and compared using the Mann–Whitney *U* test. In the association analysis between inflammatory indices and PSE, the six inflammatory indices (original values) were analyzed as continuous variables and also grouped by quartiles (Q_1_–Q_4_), with Q1 as the reference group. Three multivariable logistic regression models adjusting for different confounding factors were constructed: Model 1 was an unadjusted model; Model 2 was a basic adjustment model (adjusted for age, sex, BMI); and Model 3 was a fully adjusted model (adjusted for sex, age, smoking history, drinking history, hypertension, diabetes mellitus, cerebral infarction, coronary heart disease, BMI, systolic blood pressure, diastolic blood pressure, glycated hemoglobin, potassium, urea, creatinine, uric acid, alanine aminotransferase, aspartate aminotransferase, albumin, triglycerides, total cholesterol, homocysteine, *β*2‐microglobulin, cortical involvement, early seizures, and blood sampling time groups). Results were presented as odds ratios (ORs) with 95% confidence intervals (CIs), and trend *p* values were calculated. The Benjamini‐Hochberg method was used for false discovery rate (FDR) correction. Restricted cubic spline (RCS) models and threshold effect analyses were used to test potential nonlinear associations between the six inflammatory indices and PSE risk. ROC curves were used to calculate the area under the curve (AUC) with 95% CIs to evaluate the independent discriminative ability of each inflammatory index for PSE. Decision curve analysis (DCA) was plotted to evaluate the standardized net benefit of each index across different clinical risk thresholds. The NRI with 95% CI and IDI with 95% CI were calculated to evaluate the incremental value of adding each inflammatory index to the baseline model for PSE risk identification. Subgroup analyses stratified by sex, smoking history, drinking history, hypertension, diabetes mellitus, stroke type, and age (≤ 70 years vs. > 70 years) were performed to test the heterogeneity of the association between SIRI and PSE across different subgroups.

## 3 Results

### 3.1 Baseline Characteristics of Study Subjects

After 1:1 propensity score matching, a total of 634 patients were included (317 controls and 317 PSE cases) (Table 1). Before matching, variables including age, systolic blood pressure, diastolic blood pressure, BMI, diabetes mellitus, cerebral infarction, and coronary heart disease were imbalanced between groups; after matching, all 11 variables had SMDs < 0.13, indicating good inter‐group balance (Table S2, Figures S1, S2). There were no significant differences between the two groups in terms of sex, smoking history, drinking history, or underlying diseases (all *p* > 0.05). Regarding laboratory parameters, the case group had higher serum sodium, *β*2‐microglobulin, white blood cell count, neutrophil count, monocyte count, and aspartate aminotransferase, and lower red blood cell count, hemoglobin, albumin, and triglycerides compared with the control group (all *p* < 0.05). All six inflammatory indices were significantly elevated in the case group (all *p* < 0.001). Among them, SIRI was 0.84 (0.54, 1.22) in the control group and 1.34 (0.79, 2.29) in the case group. The case group had higher proportions of cortical involvement and early seizures (Table S1).

**TABLE 1:** Baseline characteristics of the study population after propensity score matching.

Variables	Total	Stroke	Post stroke epilepsy	*p*	SMD
	(*n* = 634)	(*n* = 317)	(*n* = 317)		
Age, Mean ± SD	72.88 ± 9.13	73.02 ± 9.04	72.74 ± 9.24	0.696	−0.031
Body Mass Index, Mean ± SD	23.61 ± 2.29	23.59 ± 2.69	23.64 ± 1.79	0.791	0.027
Systolic Blood Pressure, Mean ± SD	140.08 ± 19.57	139.51 ± 18.27	140.66 ± 20.80	0.458	0.056
Diastolic Blood Pressure, Mean ± SD	79.68 ± 12.68	78.88 ± 12.76	80.47 ± 12.56	0.114	0.127
Glycated Hemoglobin, Mean ± SD	6.44 ± 1.23	6.46 ± 1.06	6.42 ± 1.38	0.695	−0.028
Potassium, Mean ± SD	3.84 ± 0.43	3.84 ± 0.37	3.83 ± 0.48	0.804	−0.018
Sodium, Mean ± SD	141.19 ± 3.19	141.62 ± 2.41	140.77 ± 3.78	< 0.001	−0.223
Urea, Mean ± SD	5.59 ± 1.77	5.66 ± 1.62	5.52 ± 1.92	0.308	−0.075
Creatinine, Mean ± SD	75.20 ± 29.50	74.52 ± 21.53	75.89 ± 35.76	0.558	0.038
Uric Acid, Mean ± SD	323.56 ± 95.91	316.94 ± 84.84	330.17 ± 105.56	0.082	0.125
Homocysteine, Mean ± SD	13.73 ± 5.26	13.33 ± 4.90	14.12 ± 5.57	0.059	0.142
*β*2‐Microglobulin, Mean ± SD	2.71 ± 0.90	2.61 ± 0.84	2.80 ± 0.95	0.007	0.202
White Blood Cell, M (Q_1_, Q_3_)	6.21 (5.02, 7.50)	5.63 (4.78, 6.63)	6.77 (5.56, 8.45)	< 0.001	0.43
Red Blood Cell, M (Q_1_, Q_3_)	4.13 (3.77, 4.46)	4.18 (3.88, 4.51)	4.06 (3.70, 4.45)	0.006	−0.193
Hemoglobin, M (Q_1_, Q_3_)	125.00 (115.00, 135.00)	127.00 (117.00, 137.00)	123.80 (113.50, 134.00)	0.008	−0.203
Platelet Count, M (Q_1_, Q_3_)	171.50 (137.25, 212.00)	169.00 (135.00, 206.00)	177.00 (138.00, 225.00)	0.032	0.14
Absolute Neutrophil Count, M (Q_1_, Q_3_)	3.88 (2.96, 5.03)	3.38 (2.68, 4.24)	4.47 (3.42, 5.71)	< 0.001	0.462
Absolute Lymphocyte Count, M (Q_1_, Q_3_)	1.50 (1.19, 1.95)	1.51 (1.22, 1.92)	1.48 (1.16, 1.97)	0.545	−0.04
Absolute Monocyte Count, M (Q_1_, Q_3_)	0.40 (0.31, 0.51)	0.39 (0.30, 0.45)	0.47 (0.35, 0.60)	< 0.001	0.432
Alanine Aminotransferase, M (Q_1_, Q_3_)	16.10 (11.60, 22.00)	16.00 (11.60, 21.00)	17.00 (11.20, 23.00)	0.148	0.142
Aspartate Aminotransferase, M (Q_1_, Q_3_)	21.00 (16.92, 26.00)	20.00 (16.00, 24.00)	23.00 (17.00, 28.00)	< 0.001	0.252
Albumin, M (Q_1_, Q_3_)	38.61 (36.20, 41.00)	39.20 (37.30, 41.40)	37.70 (34.80, 40.10)	< 0.001	−0.467
Triglyceride, M (Q_1_, Q_3_)	1.18 (0.84, 1.67)	1.24 (0.88, 1.77)	1.14 (0.81, 1.55)	0.01	−0.237
Total Cholesterol, M (Q_1_, Q_3_)	3.88 (3.22, 4.65)	3.90 (3.22, 4.64)	3.84 (3.20, 4.65)	0.858	0.028
SIRI, M (Q_1_, Q_3_)	1.01 (0.63, 1.69)	0.84 (0.54, 1.22)	1.34 (0.79, 2.29)	< 0.001	0.527
NLR, M (Q_1_, Q_3_)	2.44 (1.77, 3.68)	2.14 (1.64, 2.92)	2.72 (2.04, 4.42)	< 0.001	0.419
Dnlr, M (Q_1_, Q_3_)	0.87 (0.84, 0.90)	0.86 (0.83, 0.89)	0.88 (0.85, 0.91)	< 0.001	0.362
MLR, M (Q_1_, Q_3_)	0.26 (0.20, 0.37)	0.24 (0.19, 0.32)	0.29 (0.22, 0.42)	< 0.001	0.416
NPR, M (Q_1_, Q_3_)	0.02 (0.02, 0.03)	0.02 (0.02, 0.03)	0.03 (0.02, 0.03)	< 0.001	0.458
NMLR, M (Q_1_, Q_3_)	2.75 (1.99, 4.00)	2.38 (1.84, 3.23)	3.08 (2.28, 4.79)	< 0.001	0.429
Sex, *n* (%)				0.808	−0.019
Female	255 (40.22)	129 (40.69)	126 (39.75)		
Male	379 (59.78)	188 (59.31)	191 (60.25)		
Smoking History, *n* (%)				0.655	−0.035
No	540 (85.17)	272 (85.80)	268 (84.54)		
Yes	94 (14.83)	45 (14.20)	49 (15.46)		
Drinking History, *n* (%)				0.556	−0.046
No	551 (86.91)	278 (87.70)	273 (86.12)		
Yes	83 (13.09)	39 (12.30)	44 (13.88)		
Hypertension, *n* (%)				0.602	−0.042
No	188 (29.65)	97 (30.60)	91 (28.71)		
Yes	446 (70.35)	220 (69.40)	226 (71.29)		
Diabetes Mellitus, *n* (%)				0.671	0.034
No	429 (67.67)	212 (66.88)	217 (68.45)		
Yes	205 (32.33)	105 (33.12)	100 (31.55)		
Cerebral Infarction, *n* (%)				0.842	0.016
No	126 (19.87)	62 (19.56)	64 (20.19)		
Yes	508 (80.13)	255 (80.44)	253 (79.81)		
Coronary Heart Disease, *n* (%)				0.66	−0.034
No	536 (84.54)	270 (85.17)	266 (83.91)		
Yes	98 (15.46)	47 (14.83)	51 (16.09)		

*Note:* Continuous variables are presented as mean ± standard deviation (Mean ± SD) for normally distributed data, or as median with interquartile range [M (Q_1_, Q_3_)] for non‐normally distributed data. Categorical variables are presented as numbers (percentages) [n (%)]. *p* values were calculated using independent samples *t*‐test for normally distributed continuous variables, Mann–Whitney *U* test for non‐normally distributed continuous variables, and chi‐square test for categorical variables. SMD, standardized mean difference.

Abbreviations: ALT, alanine aminotransferase; AST, aspartate aminotransferase; BMI, body mass index; DBP, diastolic blood pressure; DNLR, derived neutrophil‐to‐lymphocyte ratio; MLR, monocyte‐to‐lymphocyte ratio; NLR, neutrophil‐to‐lymphocyte ratio; NMLR, neutrophil–monocyte to lymphocyte ratio; NPR, neutrophil‐to‐platelet ratio; SBP, systolic blood pressure; SIRI, systemic immune‐inflammation index.

### 3.2 Logistic Regression Analysis of Inflammatory Indicators and PSE Risk

Multivariable logistic regression models were used to analyze the association between the six inflammatory indices and PSE risk, and quartile grouping was used to test dose–response relationships. Continuous variable analysis (Table 2) showed that in the unadjusted model, the basic adjustment model (age, sex, BMI), and the fully adjusted model (additionally adjusted for underlying diseases, cortical involvement, early seizures, and blood sampling time groups), all six indices were significantly positively associated with PSE risk (all FDR‐adjusted *p* < 0.001). Among them, SIRI showed the strongest association, with each one‐standard‐deviation increase associated with a 172% increase in PSE risk (OR = 2.72, 95% CI: 2.04–3.63, FDR‐adjusted *p* < 0.001); NPR was the second strongest (OR = 2.01, 95% CI: 1.60–2.53); and the ORs for the remaining indices ranged from 1.59 to 1.81 (all *p* < 0.001) (Table 2). Quartile analysis showed significant dose–response trends for all indices (all trend *p* < 0.0001). The PSE risks for the Q2, Q3, and Q4 groups of SIRI were 2.24‐fold, 2.59‐fold, and 8.75‐fold that of the Q1 group, respectively; the Q4 group of NPR had a 5.61‐fold increased risk compared with Q1; for DNLR and MLR, only the Q4 groups showed significantly increased risks (OR = 4.26 and 3.67, respectively). *E*‐value analysis showed that the *E*‐value for the SIRI‐PSE association was 4.88 (with the lower confidence limit corresponding to an *E*‐value of 3.50), and the *E*‐value for the Q4 group reached 17.0, suggesting that the association was relatively robust.

**TABLE 2:** Association between composite inflammatory markers and post‐stroke epilepsy risk in fully adjusted model with additional covariates.

Exposure	Model 1	Model 2	Model 3	
	OR (95% CI) *p* value			FDR‐*p*
SIRI (per 1‐SD increase)	2.51 (1.95, 3.23) < 0.001	2.74 (2.09, 3.59) < 0.001	2.72 (2.04, 3.63) < 0.001	< 0.001
NLR (per 1‐SD increase)	1.76 (1.44, 2.15) < 0.001	1.83 (1.48, 2.26) < 0.001	1.70 (1.36, 2.12) < 0.001	< 0.001
dNLR (per 1‐SD increase)	1.50 (1.27, 1.78) < 0.001	1.51 (1.28, 1.80) < 0.001	1.59 (1.31, 1.93) < 0.001	< 0.001
MLR (per 1‐SD increase)	1.70 (1.41, 2.05) < 0.001	1.81 (1.48, 2.21) < 0.001	1.81 (1.45, 2.26) < 0.001	< 0.001
NPR (per 1‐SD increase)	1.86 (1.52, 2.27) < 0.001	1.93 (1.57, 2.38) < 0.001	2.01 (1.60, 2.53) < 0.001	< 0.001
NMLR (per 1‐SD increase)	1.78 (1.45, 2.17) < 0.001	1.85 (1.50, 2.29) < 0.001	1.73 (1.38, 2.16) < 0.001	< 0.001
SIRI				
Q1 (Reference)	1.00 (Reference)	1.00 (Reference)	1.00 (Reference)	
Q2	1.90 (1.19, 3.01) 0.007	2.07 (1.29, 3.33) 0.002	2.24 (1.34, 3.74) 0.002	0.003
Q3	2.51 (1.58, 3.98) < 0.001	2.89 (1.79, 4.69) < 0.001	2.59 (1.53, 4.37) < 0.001	< 0.001
Q4	7.09 (4.32, 11.62) < 0.001	8.78 (5.18, 14.88) < 0.001	8.75 (4.86, 15.75) < 0.001	< 0.001
p for trend	< 0.001	< 0.001	< 0.001	< 0.001
NLR				
Q1 (Reference)	1.00 (Reference)	1.00 (Reference)	1.00 (Reference)	
Q2	1.59 (1.01, 2.50) 0.046	1.63 (1.03, 2.58) 0.036	1.62 (0.98, 2.68) 0.061	0.074
Q3	2.21 (1.40, 3.47) < 0.001	2.32 (1.46, 3.68) < 0.001	2.35 (1.42, 3.89) < 0.001	0.001
Q4	4.12 (2.58, 6.57) < 0.001	4.61 (2.84, 7.51) < 0.001	3.98 (2.33, 6.82) < 0.001	< 0.001
*p* for trend	< 0.001	< 0.001	< 0.001	< 0.001
dNLR				
Q1 (Reference)	1.00 (Reference)	1.00 (Reference)	1.00 (Reference)	
Q2	0.91 (0.58, 1.43) 0.681	0.93 (0.59, 1.46) 0.751	0.98 (0.59, 1.62) 0.931	0.963
Q3	1.31 (0.84, 2.04) 0.238	1.33 (0.85, 2.07) 0.212	1.37 (0.83, 2.25) 0.221	0.245
Q4	3.46 (2.17, 5.52) < 0.001	3.58 (2.24, 5.74) < 0.001	4.26 (2.51, 7.24) < 0.001	< 0.001
*p* for trend	< 0.001	< 0.001	< 0.001	< 0.001
MLR				
Q1 (Reference)	1.00 (Reference)	1.00 (Reference)	1.00 (Reference)	
Q2	1.13 (0.72, 1.78) 0.583	1.19 (0.76, 1.88) 0.447	1.00 (0.61, 1.65) 0.992	0.992
Q3	1.60 (1.02, 2.50) 0.039	1.75 (1.11, 2.78) 0.016	1.48 (0.89, 2.44) 0.128	0.147
Q4	3.55 (2.23, 5.64) < 0.001	4.14 (2.53, 6.77) < 0.001	3.67 (2.12, 6.35) < 0.001	< 0.001
*P* for trend	< 0.001	< 0.001	< 0.001	< 0.001
NPR				
Q1 (Reference)	1.00 (Reference)	1.00 (Reference)	1.00 (Reference)	
Q2	0.93 (0.59, 1.47) 0.762	0.97 (0.62, 1.54) 0.907	1.08 (0.65, 1.79) 0.772	0.827
Q3	1.49 (0.95, 2.32) 0.081	1.59 (1.01, 2.51) 0.046	1.81 (1.08, 3.02) 0.024	0.030
Q4	4.65 (2.88, 7.52) < 0.001	5.06 (3.08, 8.29) < 0.001	5.61 (3.20, 9.83) < 0.001	< 0.001
*p* for trend	< 0.001	< 0.001	< 0.001	< 0.001
NMLR				
Q1 (Reference)	1.00 (Reference)	1.00 (Reference)	1.00 (Reference)	
Q2	1.83 (1.15, 2.89) 0.010	1.89 (1.19, 3.00) 0.007	1.96 (1.18, 3.26) 0.009	0.012
Q3	2.67 (1.69, 4.23) < 0.001	2.87 (1.79, 4.58) < 0.001	2.96 (1.78, 4.95) < 0.001	< 0.001
Q4	4.62 (2.88, 7.40) < 0.001	5.24 (3.20, 8.57) < 0.001	4.68 (2.72, 8.07) < 0.001	< 0.001
*p* for trend	< 0.001	< 0.001	< 0.001	< 0.001

*Note:* Model 1 was an unadjusted model. Model 2 was adjusted for age, gender, and BMI. Model 3 was additionally adjusted for gender, age, smoking history, drinking history, hypertension, diabetes mellitus, cerebral infarction, coronary heart disease, BMI, systolic blood pressure, diastolic blood pressure, glycated hemoglobin, potassium, urea, creatinine, uric acid, alanine aminotransferase, aspartate aminotransferase, albumin, triglycerides, total cholesterol, homocysteine, *β*2‐microglobulin, cortical involvement, early‐onset seizures, and blood sampling time groups (≤ 6 h vs. 6–24 h). All inflammatory indicators were standardized by *Z*‐score before analysis; OR represents risk per 1‐SD increase. FDR‐adjusted *p* values were calculated using the Benjamini‐Hochberg method for Model 3.

Abbreviations: CI, confidence interval; DNLR, derived neutrophil‐to‐lymphocyte ratio; FDR, false discovery rate; MLR, monocyte‐to‐lymphocyte ratio; NLR, neutrophil‐to‐lymphocyte ratio; NMLR, neutrophil‐monocyte to lymphocyte ratio; NPR, neutrophil‐to‐platelet ratio; OR, odds ratio; SD, standard deviation; SIRI, systemic immune‐inflammation index.

### 3.3 Nonlinear Relationship and Threshold Effect Analysis Between Inflammatory Indices and PSE

**FIGURE 2:**
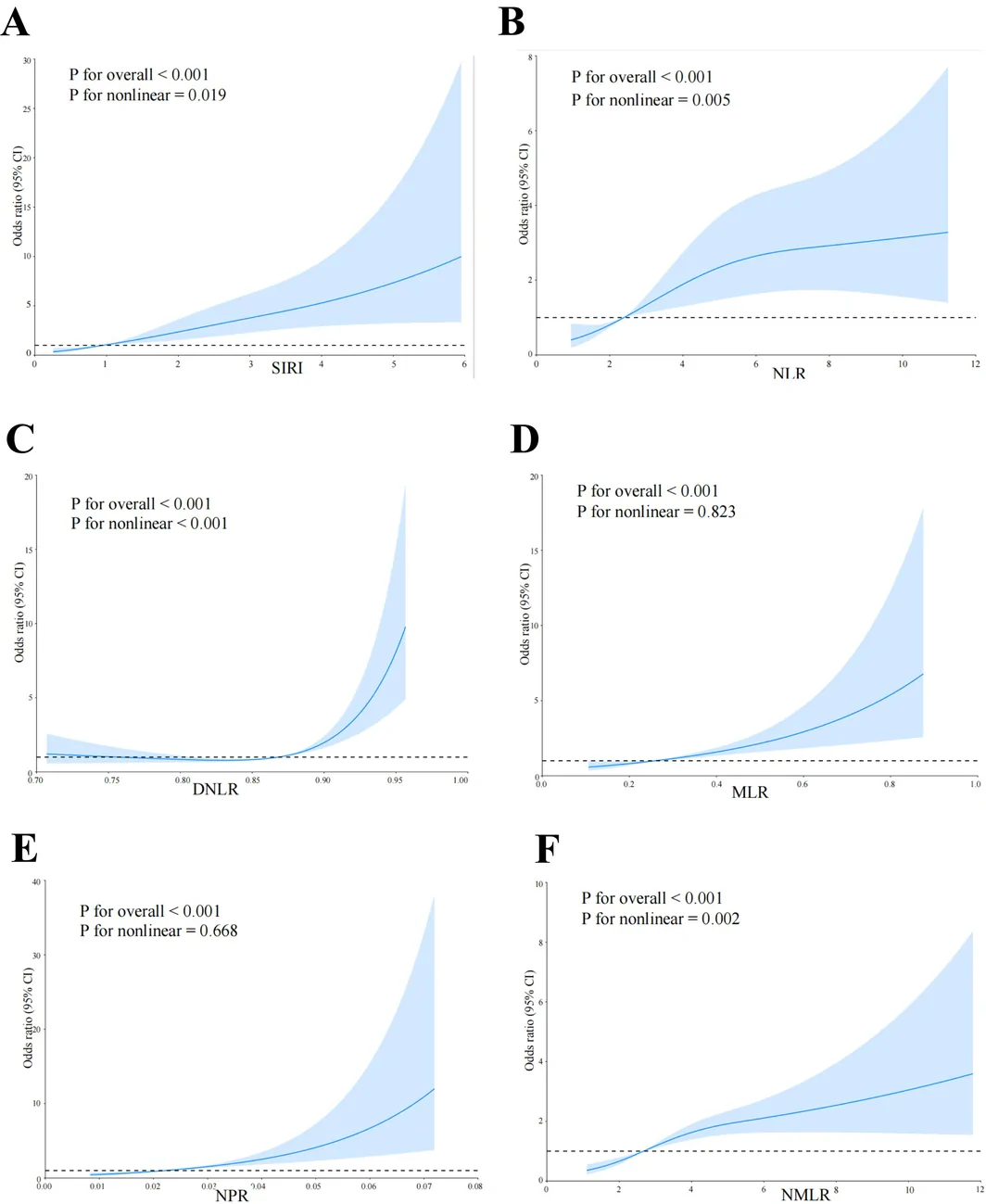
Restricted cubic spline (RCS) curves of the associations between six inflammatory indicators and post‐stroke epilepsy risk. All inflammatory indicators were *Z*‐score standardized before analysis. The shaded areas represent 95% confidence intervals. *p* for nonlinearity indicates the statistical significance of the nonlinear association. DNLR, derived neutrophil‐to‐lymphocyte ratio; MLR, monocyte‐to‐lymphocyte ratio; NLR, neutrophil‐to‐lymphocyte ratio; NMLR, neutrophil‐monocyte to lymphocyte ratio; NPR, neutrophil‐to‐platelet ratio; OR, odds ratio; SIRI, systemic immune‐inflammation index.

RCS curve analysis (Figure 2) showed that SIRI, NLR, DNLR, and NMLR exhibited nonlinear associations with PSE risk (nonlinear *p* < 0.05). Threshold effect analysis (Table 3) showed that SIRI had a significant threshold effect (Log‐likelihood ratio test *p* = 0.02), with a standardized kink point of 0.22: below the kink point, OR = 4.87 (95% CI: 2.75–8.64, *p* < 0.01); above the kink point, OR = 1.75 (95% CI: 1.17–2.64, *p* = 0.0070).

**TABLE 3:** Threshold effect analysis of multiple inflammatory indicators and PSE.

Exposure	Kink point (K)	Effect for segment < K 1	Effect for segment > K 2	Log‐likelihood ratio test
SIRI	0.22	4.87 (2.75, 8.64) < 0.01	1.75 (1.17, 2.64) 0.0070	0.02
NLR	1.60	2.48 (1.79, 3.42) < 0.01	0.77 (0.47, 1.26) 0.3011	0.00
DNLR	−0.20	0.76 (0.55, 1.05) 0.0936	3.75 (2.53, 5.56) < 0.01	< 0.001
MLR	2.12	1.92 (1.48, 2.48) < 0.01	1.04 (0.44, 2.45) 0.9246	0.25
NPR	1.61	2.42 (1.84, 3.19) < 0.01	0.97 (0.54, 1.73) 0.9166	0.03
NMLR	1.72	2.43 (1.78, 3.32) < 0.01	0.74 (0.44, 1.26) 0.2707	0.00

*Note:* All inflammatory indicators were standardized by *Z*‐score before analysis; the kink point denotes the inflection point on the *Z*‐score scale rather than raw values. Results are expressed as odds ratio (OR) with 95% confidence interval (CI). The log‐likelihood ratio test *p*‐value indicates the significance of the threshold effect.

Abbreviations: DNLR, derived neutrophil‐to‐lymphocyte ratio; K, kink point; MLR, monocyte‐to‐lymphocyte ratio; NLR, neutrophil‐to‐lymphocyte ratio; NMLR, neutrophil‐monocyte to lymphocyte ratio; NPR, neutrophil‐to‐platelet ratio; SIRI, systemic immune‐inflammation index.

### 3.4 Discriminative Performance and Added Contribution of Inflammatory Indices for PSE

**FIGURE 3:**
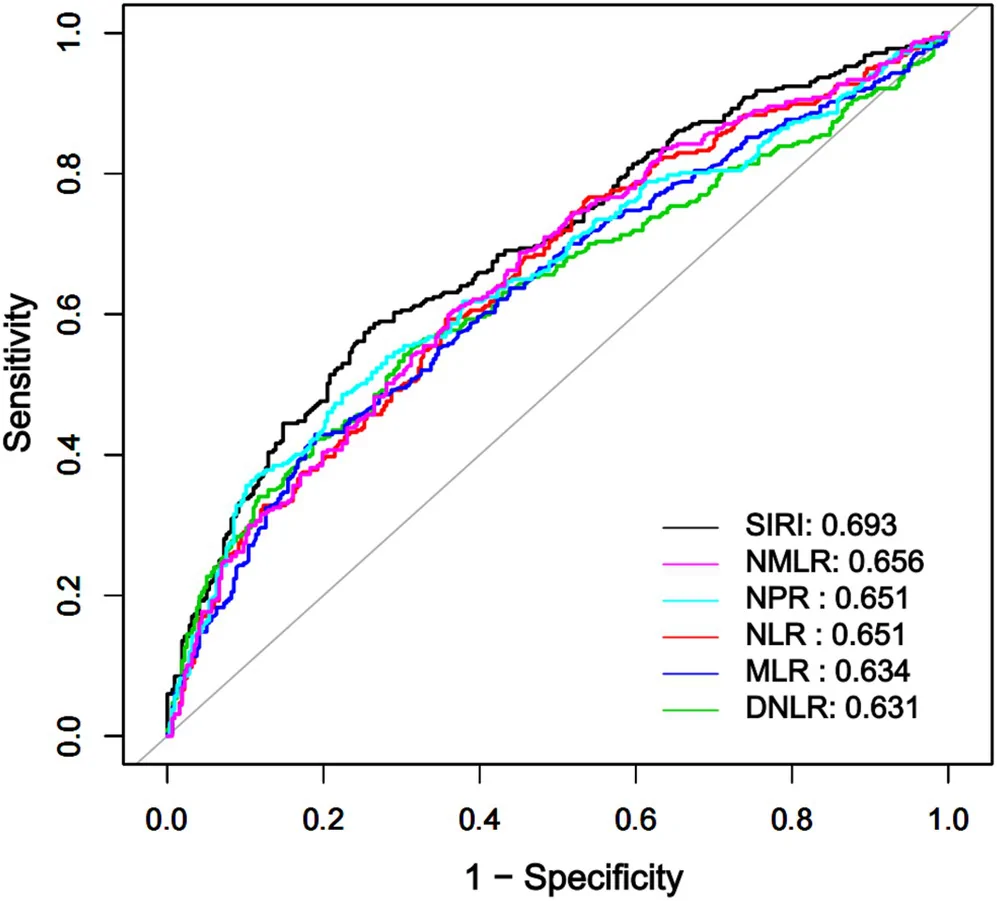
ROC curves of six inflammatory markers for discriminating PSE risk. ROC curves were generated using the original values of each inflammatory indicator; AUC, area under the curve.

**FIGURE 4:**
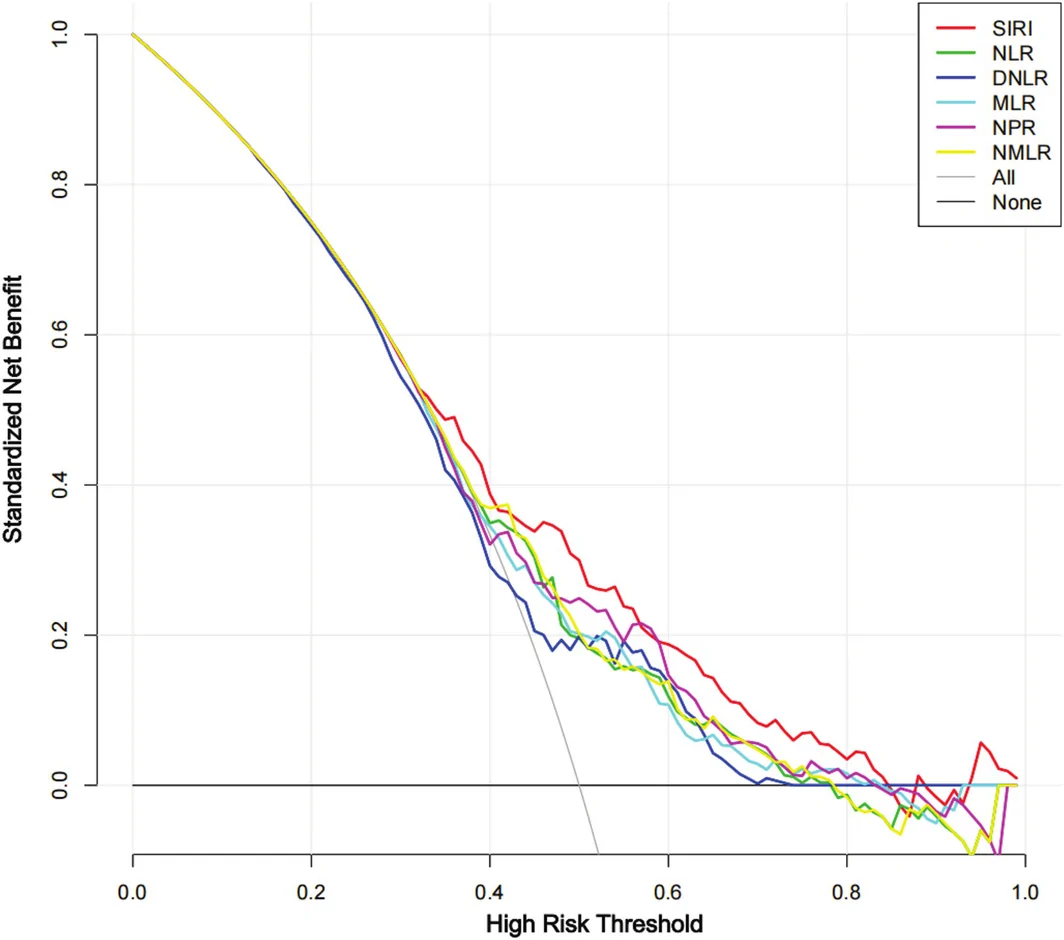
Decision curve analysis (DCA) of six inflammatory indicators for discriminating PSE risk. The horizontal axis represents the clinical risk threshold probability, and the vertical axis represents the standardized net benefit. All curves were generated using the original values of inflammatory indicators. At threshold probabilities of 10%–60%, SIRI showed a higher net benefit compared to other inflammatory indicators. The gray horizontal line represents the assumption that all patients are non‐PSE (net benefit = 0), and the gray dashed line represents the assumption that all patients are PSE. A higher net benefit at a given threshold indicates greater potential clinical utility. DNLR, derived neutrophil‐to‐lymphocyte ratio; MLR, monocyte‐to‐lymphocyte ratio; NLR, neutrophil‐to‐lymphocyte ratio; NMLR, neutrophil‐monocyte to lymphocyte ratio; NPR, neutrophil‐to‐platelet ratio; SIRI, systemic immune‐inflammation index.

ROC curve analysis based on the original values of inflammatory indicators showed that all six inflammatory indicators had a certain discriminative ability for PSE. The AUC values and 95% CIs of each indicator were as follows: SIRI AUC = 0.693 (95% CI: 0.654–0.732), NLR AUC = 0.651 (95% CI: 0.611–0.691), DNLR AUC = 0.631 (95% CI: 0.590–0.672), MLR AUC = 0.634 (95% CI: 0.593–0.675), NPR AUC = 0.652 (95% CI: 0.612–0.692), and NMLR AUC = 0.656 (95% CI: 0.616–0.696). Among them, SIRI had the highest AUC value, suggesting its relatively better discriminative performance for PSE (Table 4); the ROC curves are shown in Figure 3. Decision curve analysis (DCA) showed that all six inflammatory indicators produced positive standardized net benefit across a wide range of clinical risk thresholds. When the clinical decision threshold ranged from 10% to 60%, the net benefit of SIRI was superior to that of the other indicators; however, these DCA findings should be interpreted as hypothesis‐generating rather than evidence of direct clinical applicability, and prospective validation is needed. The DCA curves are shown in Figure 4. After adding each of the six inflammatory indicators to the baseline model, both NRI and IDI increased significantly (all *p* < 0.05). Among them, SIRI showed the most notable added contribution, with NRI = 0.1483 (95% CI: 0.0843–0.2122, *p* < 0.001) and IDI = 0.0845 (95% CI: 0.0633–0.1058, *p* < 0.001), followed by NPR, with NRI = 0.1420 (95% CI: 0.0804–0.2035, *p* < 0.001) and IDI = 0.0688 (95% CI: 0.0502–0.0875, *p* < 0.001) (Table 5).

**TABLE 4:** Discriminative ability of various inflammatory markers for PSE risk.

Test	ROC area (AUC)	Specificity	Sensitivity
SIRI	0.693	0.741	0.580
NLR	0.651	0.644	0.593
DNLR	0.631	0.688	0.552
MLR	0.634	0.811	0.429
NPR	0.652	0.776	0.486
NMLR	0.656	0.634	0.606

*Note:* All analyses were based on the original values of inflammatory indicators.

Abbreviations: AUC, area under the curve; DNLR, derived neutrophil‐to‐lymphocyte ratio; MLR, monocyte‐to‐lymphocyte ratio; NLR, neutrophil‐to‐lymphocyte ratio; NMLR, neutrophil–monocyte to lymphocyte ratio; NPR, neutrophil‐to‐platelet ratio; SIRI, systemic immune‐inflammation index.

**TABLE 5:** Incremental discriminative value of multiple inflammatory markers added to the basic model.

Variables	NRI	95% CI	*p*	IDI	95% CI	*p*
Basic model						
Basic model+SIRI	0.1483	(0.0843, 0.2122)	< 0.001	0.0845	(0.0633, 0.1058)	< 0.001
Basic model+NLR	0.0883	(0.0352, 0.1414)	0.00111	0.0382	(0.0241, 0.0523)	< 0.001
Basic model+dNLR	0.0631	(0.0022, 0.1240)	0.04225	0.0399	(0.0256, 0.0542)	< 0.001
Basic model+MLR	0.0694	(0.0120, 0.1268)	0.01789	0.0385	(0.0236, 0.0535)	< 0.001
Basic model+NPR	0.142	(0.0804, 0.2035)	< 0.001	0.0688	(0.0502, 0.0875)	< 0.001
Basic model+NMLR	0.0852	(0.0311, 0.1393)	0.00203	0.0398	(0.0254, 0.0543)	< 0.001

Abbreviations: CI, confidence interval; IDI, integrated discrimination improvement; NRI, net reclassification improvement.

### 3.5 Subgroup Analysis of SIRI and PSE Risk

**FIGURE 5:**
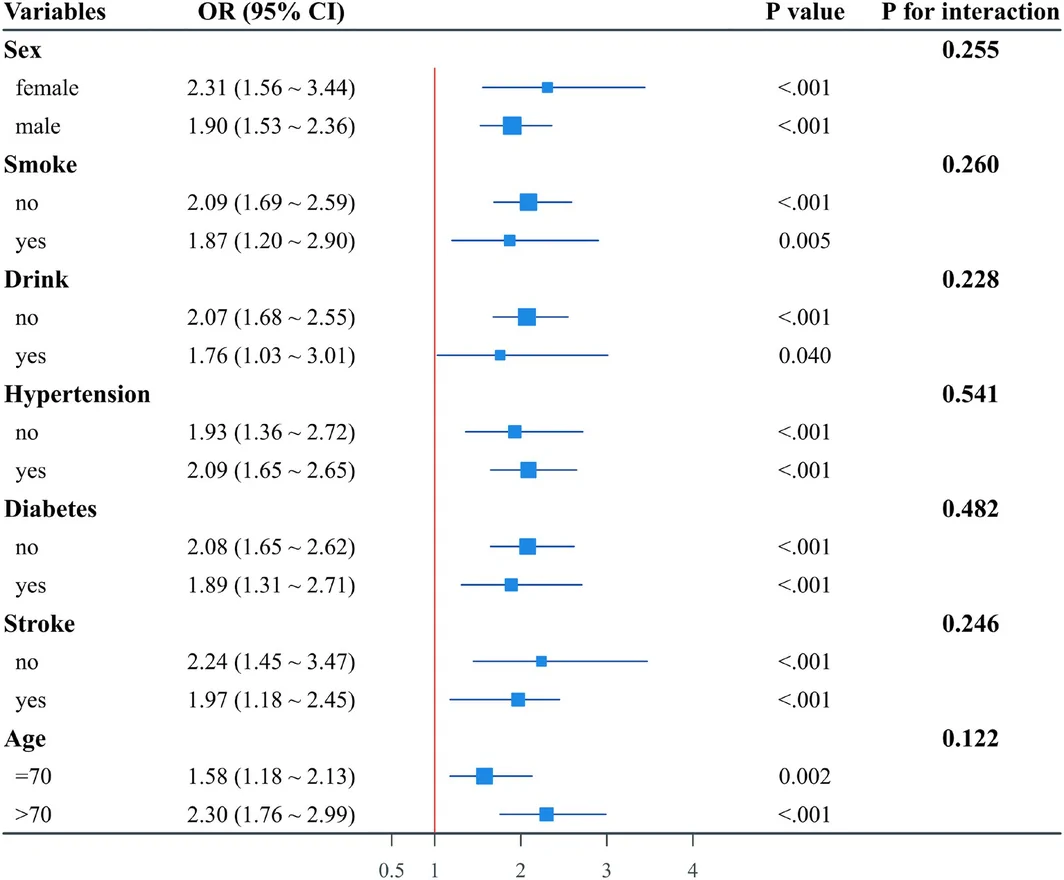
Subgroup Analysis for the Association Between SIRI and PSE Risk. Subgroup analyses were performed stratified by sex, smoking history, drinking history, hypertension, diabetes mellitus, stroke type, and age (≤ 70 years vs. > 70 years). SIRI was analyzed using original values as a continuous variable. OR, odds ratio; CI, confidence interval. P for interaction was calculated by including interaction terms between SIRI and each stratification variable in the multivariate logistic regression models, adjusted for all covariates included in Model 3. SIRI, systemic immune‐inflammation index.

Subgroup analysis was performed to explore the heterogeneity of the association between SIRI and PSE risk across different subgroups. The results showed that SIRI was significantly positively correlated with the risk of PSE in all subgroups stratified by gender, smoking history, drinking history, hypertension, diabetes mellitus, stroke type, and age (all *p* < 0.05); no significant interaction was found among the subgroups, indicating that the association of SIRI for PSE in stroke patients was stable across populations with different clinical characteristics (Figure 5).

## 4 Discussion

This study found that all six inflammation‐derived indices were significantly positively associated with PSE risk (all FDR‐adjusted *p* < 0.001), with SIRI showing the strongest association (OR = 2.72). RCS curve analysis revealed a nonlinear association between SIRI and PSE risk (nonlinear *p* < 0.05). SIRI demonstrated moderate but relatively better discriminative ability (AUC = 0.693, 95% CI: 0.654–0.732) compared with the other five indices, and DCA provided exploratory evidence of potential net benefit within threshold probabilities of 0.10–0.60, though this should be interpreted cautiously. The NRI (0.1483, 95% CI: 0.0843–0.2122, *p* < 0.001) and IDI (0.0845, 95% CI: 0.0633–0.1058, *p* < 0.001) suggested an incremental contribution to discrimination beyond traditional models; however, as exploratory metrics, they do not establish definitive clinical utility, and subgroup analyses confirmed the stability of these findings across different populations (all interaction *p* > 0.05).

Many previous studies have suggested that inflammatory response may be associated with the occurrence of post‐stroke epilepsy (PSE). A retrospective study conducted by Lin et al. in patients with primary intracerebral hemorrhage indicated that an elevated neutrophil‐to‐lymphocyte ratio (NLR) at admission was independently associated with an increased risk of PSE, with an OR of 1.861 (95% CI: 1.032–3.355, *p* = 0.039) in the high NLR group (≥ 6.3), and this association was more prominent in the subgroup with poor functional outcome; meanwhile, the combination of NLR with cortical involvement, hematoma volume and other indicators might help improve predictive efficacy (AUC = 0.841), but the study did not further explore the synergistic effect between NLR and other inflammatory indicators [10]. A retrospective study by Hu et al. focused on the relationship between the systemic immune‐inflammation index (SIRI) and PSE, and the results showed that the SIRI level in the PSE group (3.43 ± 2.74) was higher than that in the non‐PSE group (1.79 ± 1.40, *p* < 0.001); after adjusting for relevant covariates, SIRI was still positively correlated with PSE risk (OR = 1.99, 95% CI: 1.44–2.75, *p* < 0.001), and there might be a nonlinear relationship and inflection point between them (SIRI = 1.36), with a more obvious association in stroke patients with comorbidities, but the study did not compare the differences in predictive efficacy between SIRI and classic inflammatory indicators [9]. A case–control study by Saad et al. on generalized epilepsy in children observed that the proportion of peripheral blood CD4 + CD25 + highFoxp3+ regulatory T cells (Tregs) was lower in patients (1.95% ± 0.7% vs. 3.1% ± 0.9%, *p* < 0.001), while the levels of pro‐inflammatory factors such as IL‐6, TNF‐α and IL‐10 were increased, suggesting that immune imbalance might be involved in the pathological process of epilepsy in children [23]. The results of an animal experiment by Cai et al. suggested that the expression of ADAR1 was upregulated in astrocytes after ischemic stroke, which might promote astrocyte proliferation through the PI3K/Akt pathway and induce the secretion of IL‐1*β*, IL‐6 and TNF‐*α* by producing reactive oxygen species (ROS), thereby participating in neuronal apoptosis; ADAR1 deficiency might help reduce infarct volume and improve neurological function, providing experimental clues for the mechanism of inflammation involved in PSE, which needs further verification in clinical populations [24]. A two‐sample Mendelian randomization study by Sun et al. suggested that elevated circulating levels of MIF (OR = 1.077, 95% CI: 1.010–1.148, *p* = 0.024) and MIP1α/CCL3 (OR = 1.116, 95% CI: 1.004–1.241, *p* = 0.042) might be potentially causally associated with an increased risk of epilepsy, while elevated MCSF levels might have a certain protective effect (OR = 0.894, 95% CI: 0.811–0.985, *p* = 0.024), and the association between different inflammatory factors and epilepsy subtypes might vary, but the study did not focus on the stroke‐related epilepsy population [25]. Unlike previous studies, the distinctive feature of our study is the systematic analysis of six inflammation‐derived indices based on routine blood parameters. We observed that SIRI demonstrated the relatively strongest association, threshold effect analysis suggested the presence of a kink point, and it showed moderate discriminative ability (AUC = 0.693). The results remained relatively stable across subgroups of sex, age, and stroke type (all interaction *p* values > 0.05). Both NRI and IDI suggested potential incremental value when combined with traditional risk assessment models. This may provide a convenient and easily obtainable auxiliary reference indicator for early risk stratification of PSE. Nevertheless, its clinical application requires caution and urgent external prospective validation.

In recent years, several studies have suggested that peripheral blood cell counts and related inflammatory factors may play a role in the occurrence and development of post‐stroke epilepsy (PSE). Clinical studies have shown that changes in leukocytes and their subsets, as common indicators of immune inflammation, may be associated with PSE [10, 26]. One study in stroke patients observed that serum monocyte chemoattractant protein‐1 (MCP‐1) levels were higher in the epilepsy group than in the non‐epilepsy group. Logistic regression analysis suggested that MCP‐1 may be an independent risk factor for PSE, and the combined detection of MCP‐1 and endothelial cell‐specific molecule‐1 (ESM‐1) may have certain predictive value for PSE [27]. MCP‐1 is mainly secreted by monocytes and macrophages, and its elevated level may reflect the chemotaxis and activation status of monocytes to some extent [28]. A study in patients after meningioma surgery also found that white blood cell count was higher in those with new‐onset postoperative epilepsy, and the derived neutrophil/lymphocyte ratio (dNLR) was more significantly elevated in patients with persistent epilepsy, which may have certain predictive sensitivity and specificity for seizure occurrence [29]. After ischemic stroke, accumulated neutrophils can release neutrophil extracellular traps (NETs) and aggravate nerve damage through multiple inflammatory pathways. Cell division cycle 42 (Cdc42) may participate in neuroinflammation by regulating neutrophil function, thereby contributing to brain injury and epileptogenesis [28]. A comparative study suggested that after seizures, certain immune changes may occur in peripheral blood granulocyte and monocyte subsets, and the degree of alteration may be related to seizure type (e.g., generalized tonic–clonic seizures) [30]. At the genetic level, animal experiments have indicated that after status epilepticus, the mRNA expression of classic pro‐inflammatory factors such as IL‐1*β* and TNF‐*α* in the brain may be upregulated in a region‐specific manner and interact with the plasma plasminogen activation system to jointly participate in epileptogenesis [31].

The strengths of this study are mainly reflected in the following aspects. First, the use of a propensity score‐matched case–control design controlled for baseline confounding factors, contributing to the reliability of the results. Second, the comprehensive application of multidimensional statistical methods—evaluating inflammation‐derived indices from multiple perspectives including strength of association and nonlinear relationships—demonstrates scientific rigor and reference value. Third, the study population was derived from real‐world clinical practice, with a reasonably representative sample, and all index measurements used routine laboratory methods, suggesting potential for clinical application.

However, this study also has several limitations. First, as a single‐center retrospective study, the sample size was limited, and selection bias may exist; future multicenter, large‐sample prospective studies are needed for validation. In addition, the retrospective case–control design, with 1:1 propensity score matching, artificially fixed the case‐to‐control ratio at 50%, whereas the actual incidence of PSE in the real‐world stroke population is much lower. Although this artificially determined case–control ratio helped improve statistical efficiency, it may also have led to overestimation of the reported discriminative metrics such as AUC, DCA net benefit, NRI, and IDI. The results can only reflect the strength of association and discriminative ability between inflammatory indices and PSE, and cannot be directly generalized to real‐world clinical prediction scenarios. Second, the AUC value of SIRI in this study was 0.693, indicating moderate discriminative ability, which is insufficient for SIRI to serve as an independent clinical decision‐making tool. SIRI is more suitable as a component of a multi‐factor risk assessment system, used in combination with traditional clinical risk factors rather than replacing comprehensive clinical evaluation. This study did not measure specific inflammatory factors such as IL‐1*β* and TNF‐*α*, making it difficult to elucidate the quantitative relationship between SIRI and these factors or the upstream and downstream regulatory pathways. In addition, well‐recognized PSE‐related factors such as stroke severity, lesion size, hemorrhagic transformation, temporal heterogeneity, and detailed imaging subtypes could not be included in the analysis because of incomplete retrospective medical records; these unmeasured confounders may still have some influence on the results. Future research could consider expanding the sample size and conducting multicenter prospective validation to clarify the predictive value of SIRI in different populations; exploring intervention strategies using SIRI as a reference and observing the impact of anti‐inflammatory therapy on PSE incidence would provide more evidence for precise prevention and treatment of PSE.

## 5 Conclusion

This study found that six inflammation‐derived indices based on routine blood parameters were all significantly associated with the risk of developing PSE in stroke patients. Among them, the SIRI showed the strongest association (OR = 2.72, FDR‐adjusted *p* < 0.001), with a clear dose–response relationship (trend *p* < 0.001) and consistent associations across different subgroups, and its discriminative ability was relatively better among the six indices (AUC = 0.693). These findings suggest that the SIRI may serve as one of the auxiliary reference indicators for clinical risk stratification of PSE. However, the current results from DCA, NRI, and IDI should be considered exploratory rather than definitive evidence of clinical benefit, and the clinical utility of SIRI requires further validation in prospective cohort studies.

## Author Contributions

Yu Wang and Qinye Gao contributed equally to this work. They were responsible for data collection, experimental analysis, and drafting of the original manuscript. Long Wang, the corresponding author, designed the study, provided conceptual guidance, and revised the manuscript critically for important intellectual content.

## Funding

This study was supported by the Natural Science Foundation project of Bengbu Medical University (No. 2025byzd0373), the Hefei Second People's Hospital Doctoral Special Research Fund (Grant No. 2025bszx11), the Hefei Second People's Hospital Research Project (No. 2025ykt014), and the Hefei Municipal Health Commission Applied Medical Research Program (Hwk2023zd022). The funding bodies had no role in the design of the study, collection, analysis, interpretation of data, or writing of the manuscript.

## Disclosure

The authors have nothing to report.

## Ethics Statement

The principles outlined in the 2013 revision of the Helsinki Declaration were strictly complied with in this study. The Ethics Committee of the Second People's Hospital of Hefei issued an approval for the research protocol. As the present study was retrospective, our Institutional Review Board waived the written informed consent. The data of the participants would be anonymized or kept confidential, without infringing upon any of their rights and interests.

## Conflicts of Interest

The authors declare no conflicts of interest.

## Supporting information


**Table S1:** Baseline characteristics of cortical involvement, early‐onset seizures, and blood sampling time groups in the matched cohort.
**Table S2:** Covariate balance before and after propensity score matching.
**Figure S1:** Distribution of propensity scores before and after matching.
**Figure S2:** Standardized mean differences of covariates before and after propensity score matching.

## Data Availability

The data that support the findings of this study are available on request from the corresponding author. The data are not publicly available due to privacy or ethical restrictions.
