# Supplementary material for: Association Between Multiple Inflammation‐Derived Indices and the Risk of Post‐Stroke Epilepsy
Source: CNS Neurosci Ther. 2026 Sep 8;32(9):e71143. doi: 10.1002/cns.71143 (PMC13552217; doi:10.1002/cns.71143)
Supplement: Supplementary file 1 [file CNS-32-e71143-s001.docx]

Supplementary Table S1. Baseline characteristics of cortical involvement, early-onset seizures, and blood sampling time groups in the matched cohort

| Variables | Total (n = 634) | stroke  (n = 317) | Post stroke epilepsy  (n = 317) | *P* |
| --- | --- | --- | --- | --- |
|  |  |  |  |  |
| Cortical involvement, n(%) |  |  |  | 0.007 |
| No | 338 (53.31) | 186 (58.68) | 152 (47.95) |  |
| Yes | 296 (46.69) | 131 (41.32) | 165 (52.05) |  |
| Early seizures, n(%) |  |  |  | <.001 |
| No | 472 (74.45) | 296 (93.38) | 176 (55.52) |  |
| Yes | 162 (25.55) | 21 (6.62) | 141 (44.48) |  |
| Blood sampling time groups, n(%) |  |  |  | 0.661 |
| ≤6 hours | 583 (91.96) | 293 (92.43) | 290 (91.48) |  |
| 6–24 hours | 51 (8.04) | 24 (7.57) | 27 (8.52) |  |

Data are presented as n (%). P values were calculated using the chi-square test. Abbreviations: SMD, standardized mean difference.

Supplementary Table S2. Covariate balance before and after propensity score matching

| Variable | Before PSM | | | | | |  | After PSM | | | | | |
| --- | --- | --- | --- | --- | --- | --- | --- | --- | --- | --- | --- | --- | --- |
|  | Total  (n = 828) | Stroke (n = 467) | Post stroke epilepsy  (n = 361) | Statistic | P | SMD |  | Total  (n = 634) | Stroke  (n = 317) | Post stroke epilepsy  (n = 317) | Statistic | P | SMD |
| Age, Mean ± SD | 72.26 ± 9.38 | 71.42 ± 9.50 | 73.35 ± 9.12 | t=-2.952 | 0.003 | 0.212 |  | 72.88 ± 9.13 | 73.02 ± 9.04 | 72.74 ± 9.24 | t=0.391 | 0.696 | -0.031 |
| Body mass index, Mean ± SD | 23.79 ± 2.37 | 23.95 ± 2.75 | 23.60 ± 1.74 | t=2.247 | 0.025 | -0.202 |  | 23.61 ± 2.29 | 23.59 ± 2.69 | 23.64 ± 1.79 | t=-0.265 | 0.791 | 0.027 |
| Systolic blood pressure, Mean ± SD | 138.85 ± 20.50 | 135.27 ± 18.61 | 143.47 ± 21.90 | t=-5.699 | <.001 | 0.374 |  | 140.08 ± 19.57 | 139.51 ± 18.27 | 140.66 ± 20.80 | t=-0.742 | 0.458 | 0.056 |
| Diastolic blood pressure, Mean ± SD | 79.21 ± 13.04 | 77.13 ± 12.48 | 81.90 ± 13.29 | t=-5.304 | <.001 | 0.359 |  | 79.68 ± 12.68 | 78.88 ± 12.76 | 80.47 ± 12.56 | t=-1.582 | 0.114 | 0.127 |
| Gender, n (%) |  |  |  | χ²=0.970 | 0.325 |  |  |  |  |  | χ²=0.059 | 0.808 |  |
| Female | 326 (39.37) | 177 (37.90) | 149 (41.27) |  |  | 0.069 |  | 255 (40.22) | 129 (40.69) | 126 (39.75) |  |  | -0.019 |
| Male | 502 (60.63) | 290 (62.10) | 212 (58.73) |  |  | -0.069 |  | 379 (59.78) | 188 (59.31) | 191 (60.25) |  |  | 0.019 |
| Smoking history, n (%) |  |  |  | χ²=0.767 | 0.381 |  |  |  |  |  | χ²=0.200 | 0.655 |  |
| No | 705 (85.35) | 403 (86.30) | 302 (84.12) |  |  | -0.059 |  | 540 (85.17) | 272 (85.80) | 268 (84.54) |  |  | -0.035 |
| Yes | 121 (14.65) | 64 (13.70) | 57 (15.88) |  |  | 0.059 |  | 94 (14.83) | 45 (14.20) | 49 (15.46) |  |  | 0.035 |
| Alcohol history, n (%) |  |  |  | χ²=2.846 | 0.092 |  |  |  |  |  | χ²=0.347 | 0.556 |  |
| No | 727 (88.23) | 418 (89.89) | 309 (86.07) |  |  | -0.110 |  | 551 (86.91) | 278 (87.70) | 273 (86.12) |  |  | -0.046 |
| Yes | 97 (11.77) | 47 (10.11) | 50 (13.93) |  |  | 0.110 |  | 83 (13.09) | 39 (12.30) | 44 (13.88) |  |  | 0.046 |
| Hypertension, n (%) |  |  |  | χ²=3.267 | 0.071 |  |  |  |  |  | χ²=0.272 | 0.602 |  |
| No | 249 (30.15) | 152 (32.69) | 97 (26.87) |  |  | -0.131 |  | 188 (29.65) | 97 (30.60) | 91 (28.71) |  |  | -0.042 |
| Yes | 577 (69.85) | 313 (67.31) | 264 (73.13) |  |  | 0.131 |  | 446 (70.35) | 220 (69.40) | 226 (71.29) |  |  | 0.042 |
| Diabetes, n (%) |  |  |  | χ²=6.819 | 0.009 |  |  |  |  |  | χ²=0.180 | 0.671 |  |
| No | 587 (70.89) | 348 (74.52) | 239 (66.20) |  |  | -0.176 |  | 429 (67.67) | 212 (66.88) | 217 (68.45) |  |  | 0.034 |
| Yes | 241 (29.11) | 119 (25.48) | 122 (33.80) |  |  | 0.176 |  | 205 (32.33) | 105 (33.12) | 100 (31.55) |  |  | -0.034 |
| Cerebral infarction, n (%) |  |  |  | χ²=7.330 | 0.007 |  |  |  |  |  | χ²=0.040 | 0.842 |  |
| No | 191 (23.07) | 124 (26.55) | 67 (18.56) |  |  | -0.206 |  | 126 (19.87) | 62 (19.56) | 64 (20.19) |  |  | 0.016 |
| Yes | 637 (76.93) | 343 (73.45) | 294 (81.44) |  |  | 0.206 |  | 508 (80.13) | 255 (80.44) | 253 (79.81) |  |  | -0.016 |
| Coronary heart disease, n (%) |  |  |  | χ²=10.499 | 0.001 |  |  |  |  |  | χ²=0.193 | 0.660 |  |
| No | 706 (85.37) | 415 (88.87) | 291 (80.83) |  |  | -0.204 |  | 536 (84.54) | 270 (85.17) | 266 (83.91) |  |  | -0.034 |
| Yes | 121 (14.63) | 52 (11.13) | 69 (19.17) |  |  | 0.204 |  | 98 (15.46) | 47 (14.83) | 51 (16.09) |  |  | 0.034 |

Data are presented as mean ± standard deviation for continuous variables or n (%) for categorical variables. P values were calculated using independent samples t-test for continuous variables and chi-square test for categorical variables. Abbreviations: SMD, standardized mean difference; PSM, propensity score matching. After matching, all SMD values for the 11 matched variables were <0.13, indicating adequate balance between groups.

Supplementary Figure S1. Distribution of propensity scores before and after matching

The figure shows the probability density distribution of propensity scores for the treatment group (PSE, orange) and control group (stroke only, blue) before matching (upper panel) and after 1:1 nearest-neighbor matching (lower panel). After matching, the two distributions overlap substantially, indicating successful balance of the included covariates. Abbreviation: PSE, post-stroke epilepsy.

Supplementary Figure S2. Standardized mean differences of covariates before and after propensity score matching

The figure displays the absolute standardized mean differences (SMD) for each of the 11 matched covariates before (blue circles) and after (red triangles) 1:1 propensity score matching. The vertical dashed line indicates the conventional threshold for adequate balance . After matching, all covariates had SMD values below 0.13, demonstrating successful balance between the two groups. Abbreviation: BMI, body mass index; SBP, systolic blood pressure; DBP, diastolic blood pressure; SMD, standardized mean difference.
